# Draft genome sequencing of multidrug-resistant *Escherichia coli* strains isolated from mastitic cow milk

**DOI:** 10.1128/mra.01205-24

**Published:** 2025-01-29

**Authors:** Md. Morshedur Rahman, Naim Siddique, Kh. Yeashir Arafat, Syeda Fowzia Homa, Ziban Chandra Das, Tofazzal Islam, M. Nazmul Hoque

**Affiliations:** 1 Molecular Biology and Bioinformatics Laboratory, Department of Gynecology, Obstetrics and Reproductive Health, Bangabandhu Sheikh Mujibur Rahman Agricultural University (BSMRAU), Gazipur, Bangladesh; 2 Institute of Biotechnology and Genetic Engineering, BSMRAU 198780 , Gazipur, Bangladesh; University of Maryland School of Medicine, Baltimore, Maryland, USA

**Keywords:** draft genomes, *Escherichia coli*, multidrug resistance, virulence factors, mastitis, dairy cows

## Abstract

*Escherichia coli* is an important antibiotic-resistant pathogen in mastitis, with broader public health implications. We report the genomes of two *E. coli* strains, MBBL4 and MBBL5, isolated from mastitic cow milk. The draft genomes, covering 4.6 Mbp each, revealed 57.5% GC content and 100× sequencing depth.

## ANNOUNCEMENT

*Escherichia coli,* a significant gram-negative pathogen, is commonly found in food, soil, water, and various human and animal systems (1, 2). It is a leading cause of clinical mastitis (CM) in dairy cows (3, 4) and often develops antimicrobial resistance due to frequent antibiotic use (5, 6). This study reports two multidrug-resistant *E. coli* isolates from CM-affected cow milk, diagnosed via the California mastitis test (7) in Gazipur, Bangladesh (24.09° N, 90.41° E).

A 100 µL aliquot of each collected milk sample was immediately added to 10 mL of nutrient broth (Biolife, Italy) and incubated overnight at 37°C (3). The enriched broth (2 µL) was then spread onto eosin-methylene blue (EMB, Scharlau, Spain) agar and incubated at 37°C for 24 hours. Presumptive colonies were subsequently subcultured on EMB agar for purification. The phenotypic characterization of *E. coli* isolates was conducted based on colony morphology (characterized by a green metallic sheen and circular shape), Gram staining (indicating gram negative), and various biochemical tests (3, 5). The VITEK-2 system version 9.01 (8) was used to predict the species-level identification of the isolates. The DNA extraction and whole-genome sequencing were performed following our previously published protocols (9, 10). Briefly, QIAamp DNA Mini Kit (Qiagen, Germany) was used for DNA extraction from the cultured broth of the *E. coli* isolates, and libraries were prepared from 1 ng of DNA using the Nextera DNA Flex Kit (Illumina, USA). Thereafter, sequencing was executed on an Illumina MiSeq platform, with a 2 × 250 bp protocol (11). Trimmomatic version 0.39 (12) (k = 20, mink = 6, ktrim = r, ftm = 5, qtrim = rl, trimq = 20, minlen = 30, overwrite = true) (13) was employed to remove Illumina adapters, known Illumina artifacts, and phiX, and quality was checked using the FastQC version 0.11.7 (14). Thereafter, reads with phred scores of >20 were assembled using SPAdes version 3.15.5 (15), followed by annotation with the NCBI Prokaryotic Genome Annotation Pipeline version 6.6 (16). Furthermore, genome completeness was calculated by CheckM version 1.2.3 (17). In the assembled genomes, antibiotic resistance genes (ARGs) were predicted by CARD (18), plasmids by PlasmidFinder (19), and virulence factor genes (VFGs) by VFDB (20) and ecoli_VF, bundled in ABRicate (https://github.com/tseemann/abricate) on Linux command line program. The RAST version 2.0 (21) web server was used to identify the number of subsystems, whereas PathogenFinder version 1.1 (22) was used to predict the probability of the strains being human pathogens. Default parameters were used for all software unless otherwise specified.

The general genome features of *E. coli* MBBL4 and MBBL5 are given in Table 1. The assembled genomes were ~4.6 Mbp with 57.5% GC content and shared 98.50% ANI. MBBL4 contained 49 ARGs, while MBBL5 had 42, conferring resistance to aminoglycosides, fluoroquinolones, and tetracyclines. MBBL5 exhibited more VFGs (VFDB = 86; ecoli_VF = 185) than MBBL4 (VFDB = 53; ecoli_VF = 139). Both genomes contained 376 subsystems involved in specific biological processes and were identified as human pathogens, with PathogenFinder scores of 0.857 for MBBL4 and 0.887 for MBBL5.

**TABLE 1:** Genomic features of *E. coli* strains, MBBL4 and MBBL5, isolated from raw milk of mastitic cows

Genomic features	*E. coli* strains	
	MBBL4	MBBL5
GenBank accession	JBINJR000000000	JBINJQ000000000
BioSample accession	SAMN44249571	SAMN44249572
SRA accession	SRR30951065	SRR30951064
Assembled genome size (bp)	4,561,349	4,600,128
No. of reads generated	756,463	991,131
GC%	50.5	51
Coverage (×)	100	100
Average nucleotide identity (%)	98.50	98.50
Genome completeness (%)	98.53 (0.27% contamination)	99.56 (0.7% contamination)
*N*_50_ value (bp)	110,283	194,709
Number of contigs	125	71
Genes (total)	4,469	4,448
Coding sequences (CDSs) (total)	4,398	4,385
Genes (coding)	4,196	4,277
CDSs (with protein)	4,196	4,277
Genes (RNA)	71	63
rRNAs	2, 1, 2 (5S, 16S, 23S)	2, 1, 1 (5S, 16S, 23S)
Complete rRNAs	2, 1, 1 (5S, 16S, 23S)	1, 1 (16S, 23S)
tRNAs	54	52
ncRNAs	12	7
Pseudogenes (total)	202	108
CDSs (without protein)	202	108
Pseudogenes (ambiguous residues)	0 of 202	0 of 108
Pseudogenes (frameshifted)	64 of 202	53 of 108
Pseudogenes (incomplete)	129 of 202	61 of 108
Pseudogenes (internal stop)	47 of 202	21 of 108
Pseudogenes (multiple problems)	36 of 202	26 of 108
CRISPR arrays	2	2
Number of ARGs	49	42
Number of VFGs	VFDB = 53; ecoli_VF = 139;	VFDB = 86; ecoli_VF = 185;
Number of subsystems	376	376
PathogenFinder score	0.857	887
Matched with pathogenic families	136	155

## Data Availability

The whole genome shotgun project of the *Escherichia coli* strains has been deposited in GenBank and the NCBI Sequence Read Archive (SRA) under BioProject accession number PRJNA1171635. The versions described in this paper are version JBINJR000000000.1 and JBINJQ000000000.1.
